# Bilateral Bell’s Palsy With Herpes Zoster Virus

**DOI:** 10.7759/cureus.73878

**Published:** 2024-11-17

**Authors:** Punam Palit, Nayab Muneeruddin Shaik, Blessy Daniel, Ahmed S Al-Jizani, Nkechi Achufusi

**Affiliations:** 1 Medicine, Peterborough City Hospital, Peterborough, GBR; 2 Internal Medicine, Northwest Anglia NHS foundation Trust, Peterborough, GBR; 3 Internal Medicine, Royal Devon University Healthcare NHS Foundation Trust, North Devon, GBR; 4 Gastroenterology, Peterborough City Hospital, Peterborough, GBR; 5 Internal Medicine, Peterborough City Hospital, Peterborough, GBR

**Keywords:** all neurology, bell's palsy, bilateral bell's palsy, bilateral facial weakness, varicella-zoster (chickenpox), varicella-zoster virus

## Abstract

Bilateral Bell’s palsy is a rare neurological condition characterized by the sudden onset of facial nerve paralysis on both sides of the face. Unlike the more common unilateral Bell’s palsy, bilateral cases are often associated with underlying systemic or infectious causes, including viral infections. Herpes zoster virus, known for causing shingles, is a notable viral pathogen linked to facial nerve paralysis, primarily due to its ability to reactivate from latency within sensory ganglia. Although herpes zoster typically presents with a unilateral rash and nerve involvement, its atypical reactivation can lead to bilateral facial nerve involvement in immunocompetent or vulnerable individuals. This case report presents a rare instance of bilateral Bell’s palsy in a patient who had recently completed treatment for a herpes zoster infection. It includes a discussion on herpes zoster and its association with bilateral Bell’s palsy.

## Introduction

Bell's palsy is a neurological disorder characterized by sudden, unilateral facial nerve (cranial nerve VII) paralysis, typically of idiopathic origin. It is a common condition affecting one side of the face, but in rare instances, it can present bilaterally [1]. Bilateral Bell's palsy, defined as facial paralysis on both sides, accounts for around 2% of all facial nerve paralysis cases, affecting about 1 in 5,000,000 people [2]. Its presentation is often linked to underlying systemic conditions, including infections, autoimmune diseases, and neurological disorders [3]. The rarity of bilateral Bell's palsy presents a diagnostic challenge, requiring a thorough evaluation to exclude other potential causes of bilateral facial paralysis, such as Guillain-Barré syndrome, Lyme disease, and sarcoidosis [4]. Herpes zoster virus, a significant infectious agent associated with bilateral facial paralysis, can reactivate from latent states in cranial nerve ganglia, causing inflammation and nerve damage [5]. Most patients with bilateral facial paralysis often have underlying medical conditions, and bilateral facial palsy may be the initial presenting symptom. Consequently, comprehensive evaluation through laboratory tests and radiological imaging is typically essential [6]. However, in cases where the varicella-zoster virus is clearly identified as the cause, as demonstrated by the characteristic chickenpox rash, extensive laboratory investigation may not be necessary. This introduction explores the mechanisms underlying bilateral Bell's palsy, with a particular emphasis on its association with the herpes zoster virus.

## Case presentation

Medical history and demographics

A 33-year-old man presented to the emergency department with a two- to three-day history of intermittent bitemporal headaches, difficulty closing his left eye, drooping of the left eyelid, and slurred speech. Five days prior, he had initially presented with a strange sensation on the right side of his face, insomnia, difficulty rinsing his mouth as water trickled out, and an inability to properly close his right eye, along with right auricular and posterior auricular pain. He also reported difficulty chewing, drooling on the right side, numbness and tingling on the right side of his face, a change in taste, and difficulty with articulation over the past five days. He exhibited symptoms of right-sided facial muscle weakness, including drooping of the right side of the mouth, loss of the nasolabial fold, and an inability to close his right eye. He was able to swallow and denied any dysphagia. Blood tests were unremarkable, and vital signs were normal. A diagnosis of right Bell's palsy was made, and he was discharged with oral acyclovir and prednisolone 60 mg.

**Figure 1:**
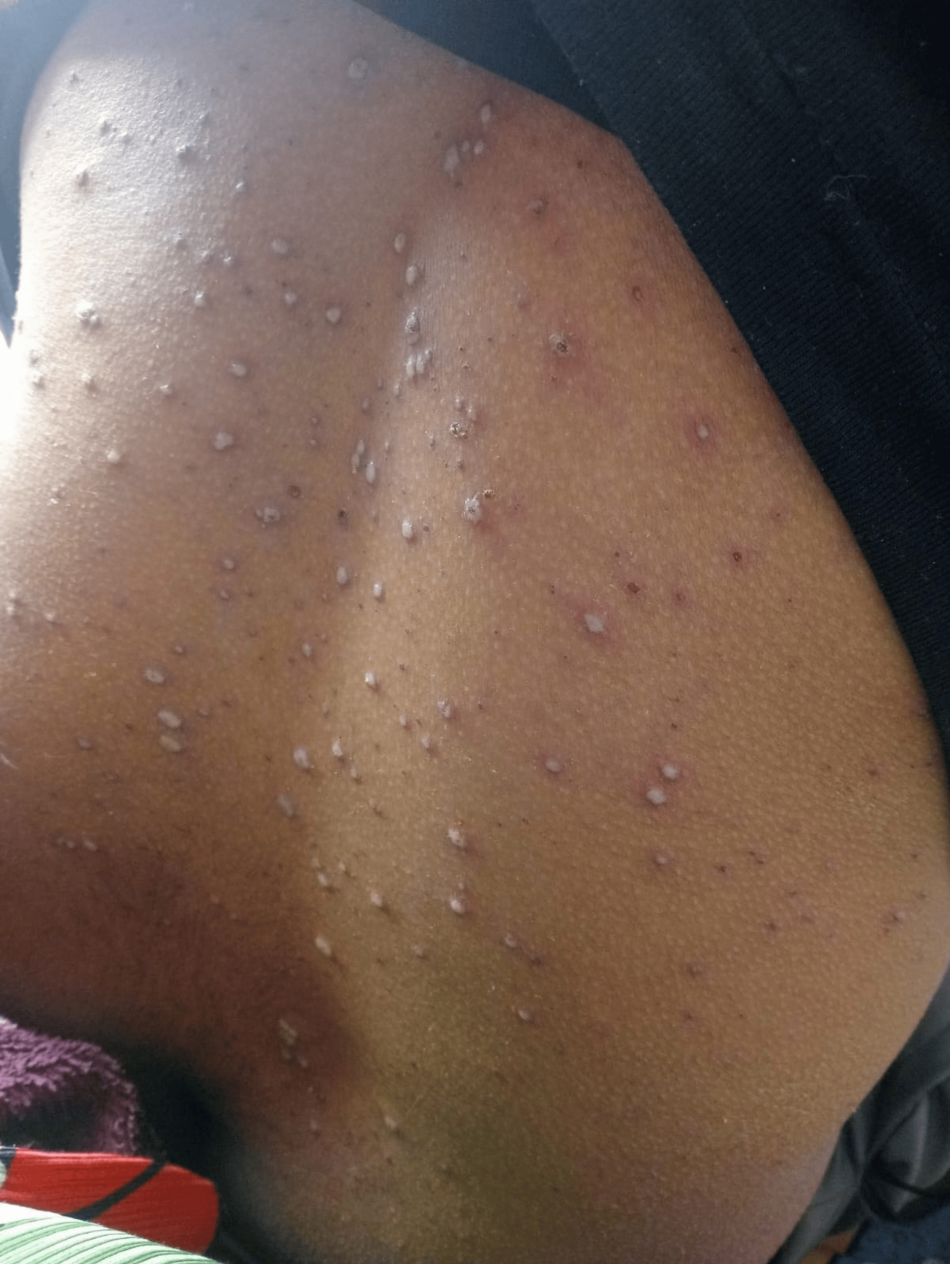
Characteristics of chicken pox rash at different stages: small red spots turned into fluid-filled blisters, which then began to scab, with some blisters already scabbed

Following reassessment, the patient exhibited bilateral facial droop and lid lag, an inability to blow out his cheeks and show his teeth, an inability to wrinkle his forehead, and an inability to close both eyes. His facial sensation was normal. He had a full range of eye movements, normal visual fields, normal movement of the tongue and palate, and normal power, coordination, and sensations in all limbs. Reflexes were present, and there was no evidence of meningitis or encephalitis. The patient’s bilateral facial nerve palsy had started on the right side and then developed on the left. He also had a widespread rash with vesicles that were scabbed over, normal vital signs, and no significant past medical history apart from a recent varicella-zoster infection that led to chickenpox (Figure 1). He was not on any medications, worked as a retail supervisor, was a non-smoker and non-alcoholic, and denied any drug use.

Investigation

Initial blood tests showed a slightly raised white cell count of 12.6 x 109/L, C-reactive protein levels of 1 mg/L, normal urea and electrolytes, creatinine 82 mmol/L, an estimated glomerular filtration rate of >90 ml/min, and normal liver enzymes. The human immunodeficiency virus screen was negative (Table 1).

**Table 1:** Results of blood investigations done at the emergency department Bloods showed slightly raised white blood cell count and slightly raised alanine transaminase, otherwise normal results.

Investigation	Result	Normal range
C-reactive protein	<1	<5 mg/L
White cell count	12.6	4.0-11.0 x 10^9^/L
Haemoglobin	148	130-180 g/L
Platelets	291	150-400 x 10^9^/L
Red blood cells	5.74	4.4-6.5 x 10^12^/L
Haematocrit	0.443	0.400-0.530 L/L
Neutrophils	10.3	1.8-7.7 x 10^9^/L
Lymphocytes	1.7	1.4-4.8 x 10^9^/L
Monocytes	0.5	0.1-0.8 x 10^9^/L
Eosinophils	0	0.1-0.6 x 10^9^/L
Basophils	0.1	0-0.1 x 10^9^/L
Albumin	45	35-50 g/L
Total bilirubin	5	0-21 umol/L
Alanine transaminase	46	<41 IU/L
Alkaline phosphatase	106	30-130 IU/L
Total protein	71	60-80 g/L
Sodium	137	133-146 mmol/L
Potassium	4.4	3.5-5.3 mmol/L
Chloride	104	95-108 mmol/L
Creatinine	82	59-104 mmol/L
eGFR result	>90	>60 ml/min
Urea	3.9	2.5-7.8 mmol/L
Human immunodeficiency virus	Negative	Positive/negative

**Figure 2:**
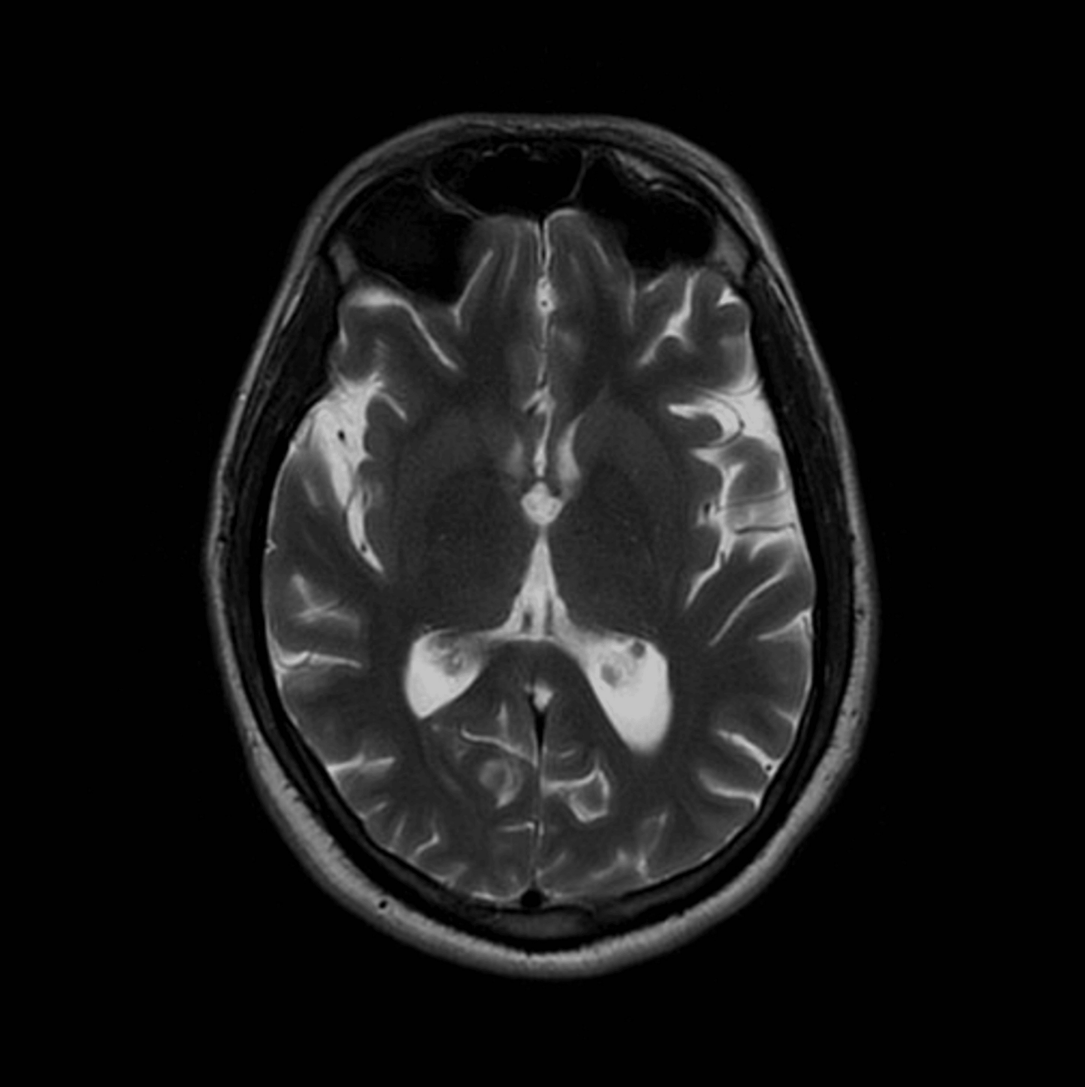
A magnetic resonance imaging scan with contrast of head Showing normal scan, no intracranial pathology was identified.

A magnetic resonance imaging scan of the head with contrast performed the following day revealed a prominent ventricular system and cortical sulci, greater than expected for the patient’s age. It did not show cerebral infarction, acoustic nerve tumor, or cerebellar atrophy. A magnetic resonance imaging scan of the brain was normal, thereby ruling out central lesions causing facial palsy (Figure 2). A diagnosis of post-varicella-zoster virus infection bilateral facial palsy was established.

Treatment

The patient was discharged with a tapering dose of prednisolone 60 mg once daily, a full one-week course of acyclovir 800 mg five times daily, and carmellose sodium 0.5% eye drops every two hours for eye care.

Outcome and follow-up

At a follow-up appointment in the neurology clinic four months later, the patient reported significant improvement in his symptoms over the course of a month. He now experiences minimal residual effects, including very subtle left-sided difficulty in sealing his mouth and a slightly reduced blink on the left with Bell’s phenomenon, neither of which cause him any significant issues.

## Discussion

In this case report, we presented a rare instance of bilateral Bell’s palsy associated with varicella-zoster virus infection. Simultaneous palsy involves the opposite side within 30 days of the first side's onset, while recurrent alternated palsy refers to contralateral facial palsy occurring more than 30 days after the first side [7]. The patient exhibited facial weakness initially on the right side then rapid onset to the left side, which was confirmed through clinical examination and a typical chickenpox rash diagnosed 10 days ago. Initially, a lumbar puncture was planned to check for varicella-zoster virus DNA in the cerebrospinal fluid, but we did not proceed to prevent the patient from undergoing a painful and uncomfortable procedure, given the clear cause of the typical rash. The patient responded well to a combination of antiviral therapy (acyclovir) and corticosteroids (prednisone), leading to significant improvement in facial function. This case highlights the importance of considering varicella-zoster virus as a potential etiological factor in bilateral Bell’s palsy, especially in immunocompetent individuals. Early recognition and prompt treatment are crucial for optimal recovery and prevention of long-term complications.

The incidence of bilateral facial palsy varies by region, with central and eastern European countries reporting the highest rates in Europe [8]. Bell’s palsy is classified into five categories: unilateral nonrecurrent, unilateral recurrent, simultaneous bilateral, alternating bilateral, and recurrent bilateral [9]. There are several causes of facial nerve palsy, including viral or bacterial infections like Lyme disease, post-influenza, infectious mononucleosis, human immunodeficiency virus, poliomyelitis, neoplastic causes like acute leukemia, acoustic neuroma, traumatic causes include skull fractures, parotid surgery, mastoid surgery, neurological causes include multiple sclerosis, pseudobulbar and bulbar palsy, Parkinson’s disease, and metabolic causes include diabetes, acute porphyria, and idiopathic factors (Table 2). Among these, autoimmune causes account for the majority of cases, followed by infections, idiopathic, traumatic, and neoplastic causes [10]. In contrast to unilateral facial nerve palsy, where idiopathic is the most common cause, accounting for up to 50%, no further investigation is required [10]. Bilateral facial nerve palsy is usually caused by an underlying systemic disease and requires workup including imaging, blood tests, and neurotological or neurological consultation. However, we have presented a case involving a 33-year-old with bilateral Bell’s palsy, directly attributed to the varicella-zoster virus. Bell’s palsy, attributed to the reactivation of latent herpes virus infection, has an incidence ranging from 13 to 34 per 100,000 individuals [5]. This is a compelling report with a clear causative factor. Below are the causes of acquired bilateral facial palsy.

**Table 2:** Causes of acquired bilateral peripheral facial palsy [10]

Differential diagnosis	Associated conditions
Trauma	Skull fractures, parotid surgery, mastoid surgery
Infection	Post influenza, infectious mononucleosis, human immunodeficiency virus, infection, Lyme disease, Banwarth’s syndrome, Guillain–Barre syndrome, syphilis, brainstem encephalitis, human T-lymphotropic virus type 1, poliomyelitis
Metabolic	Diabetes, acute porphyria
Neoplastic	Acute leukemia, acoustic neuroma
Autoimmune	Sarcoidosis, amyloidosis
Neurological	Multiple sclerosis, pseudobulbar and bulbar palsy, Parkinson’s disease
Idiopathic	Bell’s palsy

For diagnosing facial nerve palsy, a good clinical history and physical examination are sufficient. So, it is always important to include onset, associated symptoms, any recent illness, and relevant risk factors like viral prodrome, vesicles, rashes, and immunization. It’s always important to keep differentials broad and rule out any life-threatening conditions if suspected. Rapid-onset palsy is often triggered by infections or autoimmune disorders, while slowly progressive palsy may indicate neoplastic diseases [2].

Our patient’s treatment and subsequent improvement align with existing literature, which underscores the efficacy of combined antiviral and corticosteroid therapy [7]. At follow-up, the patient reported minimal residual effects, highlighting the importance of early intervention.

This case adds to the limited body of knowledge on bilateral Bell’s palsy by emphasizing the necessity of considering the herpes zoster virus in differential diagnoses, particularly in immunocompetent individuals. The clinical significance of this report lies in its demonstration of the need for thorough evaluation and prompt treatment, which are vital for optimal recovery and prevention of long-term complications.

## Conclusions

This case report describes a rare occurrence of bilateral Bell’s palsy resulting from varicella-zoster virus in an immunocompetent patient. The patient’s dual facial paralysis was clinically diagnosed based on the presence of a typical chickenpox rash, without the need for laboratory confirmation. Immediate treatment with antiviral medication (acyclovir) and corticosteroids (prednisone) led to significant recovery. This case highlights the importance of recognizing varicella-zoster virus as a potential cause of bilateral Bell’s palsy. Employing a thorough diagnostic approach and consulting neurology urgently is essential to ensure favorable outcomes and prevent long-term complications.
